# Etiology and treatment of adrenoleukodystrophy: new insights from *Drosophila*

**DOI:** 10.1242/dmm.031286

**Published:** 2018-06-15

**Authors:** Hannah B. Gordon, Lourdes Valdez, Anthea Letsou

**Affiliations:** Department of Human Genetics, University of Utah, Salt Lake City, UT 84112, USA

**Keywords:** Bubblegum (Bgm), Double bubble (Dbb), ABCD1, Fatty acid acyl-coA synthetase, Fatty acid transporter, Elongase, Neurodegeneration

## Abstract

Adrenoleukodystrophy (ALD) is a fatal progressive neurodegenerative disorder affecting brain white matter. The most common form of ALD is X-linked (X-ALD) and results from mutation of the *ABCD1*-encoded very-long-chain fatty acid (VLCFA) transporter. X-ALD is clinically heterogeneous, with the cerebral form being the most severe. Diagnosed in boys usually between the ages of 4 and 8 years, cerebral X-ALD symptoms progress rapidly (in as little as 2 years) through declines in cognition, learning and behavior, to paralysis and ultimately to a vegetative state and death. Currently, there are no good treatments for X-ALD. Here, we exploit the *Drosophila bubblegum* (*bgm*) *double bubble* (*dbb*) model of neurometabolic disease to expand diagnostic power and therapeutic potential for ALD. We show that loss of the *Drosophila* long-/very-long-chain acyl-CoA synthetase genes *bgm* and/or *dbb* is indistinguishable from loss of the *Drosophila* ABC transporter gene *ABCD*. Shared loss-of-function phenotypes for synthetase and transporter mutants point to a lipid metabolic pathway association with ALD-like neurodegenerative disease in *Drosophila*; a pathway association that has yet to be established in humans. We also show that manipulation of environment increases the severity of neurodegeneration in *bgm* and *dbb* mutant flies, adding even further to a suite of new candidate ALD disease-causing genes and pathways in humans. Finally, we show that it is a lack of lipid metabolic pathway product and not (as commonly thought) an accumulation of pathway precursor that is causative of neurometabolic disease: addition of medium-chain fatty acids to the diet of *bgm* or *dbb* mutant flies prevents the onset of neurodegeneration. Taken together, our data provide new foundations both for diagnosing ALD and for designing effective, mechanism-based treatment protocols.

This article has an associated First Person interview with the first author of the paper.

## Abstract (precis)

**Summary:** Using a *Drosophila* model of adrenoleukodystrophy, the authors provide evidence against the current view that an accumulation of lipid metabolic pathway precursors is causative of this neurometabolic disease. Rather, a lack of pathway product is the causative factor and addition of medium-chain fatty acids to the diet prevented the onset of neurodegeneration.

## INTRODUCTION

Adrenoleukodystrophy (ALD) is a neurodegenerative disorder associated with mutation of the peroxisomal ABC transporter protein ALDP (adrenoleukodystrophy protein) that is encoded by the X chromosome locus *ABCD1* (Mosser et al., 1993)*.* The spectrum of clinical features associated with ALDP mutation is broad, ranging from adrenocortical insufficiency to slowly progressive myelopathy to cerebral demyelination (Raymond et al., 1999). ALDP is required for transport of very-long-chain fatty acids (VLCFAs), and ALDP deficits lead to VLCFA accumulation in plasma and tissue (Engelen et al., 2012; Raymond et al., 1999). With respect to disease etiology, it is thought that VLCFA accumulation is toxic to the adrenal gland and to the myelin sheath that surrounds the many nerve cells of the body (Engelen et al., 2012). However, several inconsistencies exist in patient studies that refute this model. First, while all individuals harboring disease-associated alleles of *ABCD1* exhibit VLCFA level increases, some never manifest neurodegenerative symptoms (Engelen et al., 2012; Raymond et al., 1999). Second, VLCFA levels do not correlate with patient neurological disabilities (Moser, 1997). Third, although the two recipients of hematopoietic stem cell therapy showed improvement in their neurological symptoms, plasma VLCFA concentrations remained high (Cartier et al., 2009). In addition to the documented role for the ABC transporter in VLCFA metabolism and ALD, a role for the acyl-CoA synthases (ACSs) that function immediately upstream of ABC transporters in fatty acid (FA) metabolism has long been contemplated, as decreased ACS activity is another biochemical hallmark of ALD (Hashmi et al., 1986; Lazo et al., 1988; Wanders et al., 1988). Consistent with this idea is the recent identification of a patient with ALD-like cerebral degeneration with a rare mutation in the *SLC27a6*-encoded ACS (Sivachenko et al., 2016).

The cerebral form of ALD is severely progressive and, in the absence of cures and treatments, death is inevitable. It is also clear that our current understanding of ALD disease etiology is insufficient for the design of effective treatment protocols; in this regard, therapeutic manipulation of VLCFA levels does not impact disease progression (Engelen et al., 2012; Raymond et al., 1999). Animal models of neurometabolic disease, however, continue to enhance our understanding of ALD and yield new insights into ALD diagnosis and management. In particular, neurometabolic disease models in the mouse and fly are consistent with roles for ACSs in ALD (Heinzer et al., 2003; Min and Benzer, 1999; Sivachenko et al., 2016). These genetic platforms offer new opportunities for: (1) dissecting ALD-associated pathways enhancing early ALD diagnostic power and (2) identifying molecular targets suitable for therapeutic inhibition as well as alternative pathways that can potentially be boosted to alleviate degeneration.

## RESULTS

### Loss of neuronal *Drosophila ABCD* transporter function causes neurodegeneration

**Fig. 1.**
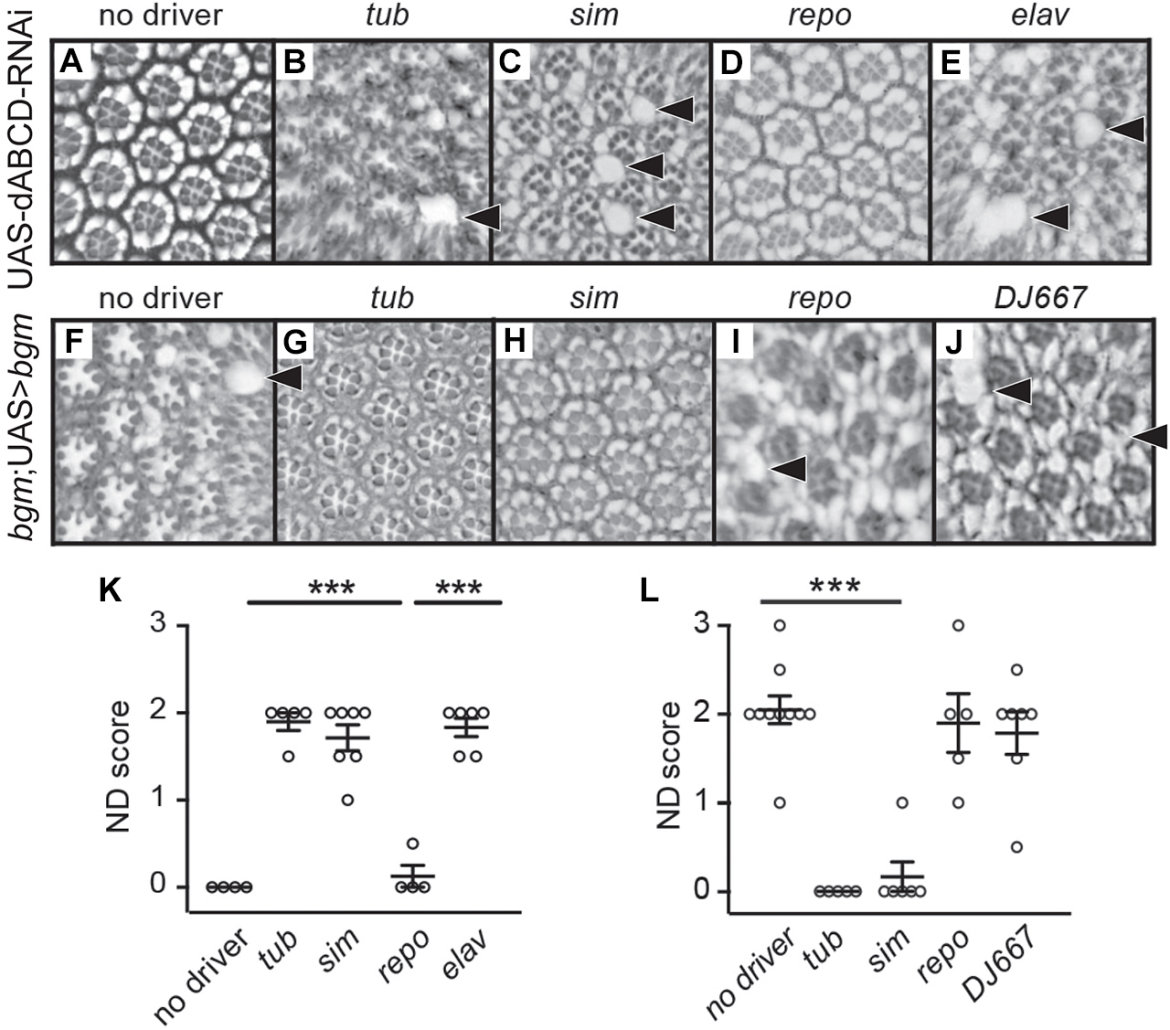
***dABCD* and *bgm* are required in adult retinal neurons.** (A) Retinal cross-sections of UAS-*dABCD*-RNAi control animals (no *Gal4* driver) reveal a highly organized ommatidial structure. (B) In contrast, ubiquitous knockdown of *dABCD* results in a shared loss-of-function phenotype with *bgm* and *dbb* mutants, with holes and disrupted pigment cells between ommatidia. (C,E) Neuronal knockdown of *dABCD*, but not glial knockdown (D), also leads to neurodegeneration. (F) Representative cross-section of a *bgm* mutant at day 20 post-eclosion. (G) Ubiquitous expression (*tub*-Gal4) of an inducible *bgm*+ transgene rescues neurodegeneration in a *bgm* mutant background. (H) Driving *bgm*+ expression in neuronal cells (*sim*-Gal4) is sufficient to rescue the mutant phenotype; however, (I) driving *bgm*+ in glial cells (*repo*-Gal4) does not result in rescue. (J) *DJ667*-Gal4, which was used as a negative control as it is specifically expressed in flight muscles, fails to rescue. (K) Blinded quantification of neurodegenerative (ND) phenotypes in A-E. (L) Blinded quantification of neurodegenerative phenotype seen in G-J. In K and L, scores were compared by one-way ANOVA with Dunnett's test and *P*-values. Data points represent biological replicates and are presented with means±s.e.m. ***P<0.001. Arrowheads point to areas of retinal degeneration.

Although neurodegeneration reminiscent of ALD has been successfully modeled in ACS loss-of-function flies (Min and Benzer, 1999; Sivachenko et al., 2016), the analysis of orthologs of the X-linked ALD (X-ALD) human disease gene (*ABCD1*) in animal models has remained elusive (Kobayashi et al., 1997) – in vertebrates likely due to gene duplication. This said, the recent development of comprehensive and bioinformatically validated RNAi libraries facilitating reverse genetic approaches has opened a pipeline for gene validation in the non-redundant (less highly duplicated) genome of the fly (Moulton and Letsou, 2016 for review). Using reciprocal BLASTp algorithms, we identified *CG2316* as the sole *Drosophila* homolog of human *ABCD1*. *CG2316*, a fourth chromosome gene henceforth designated *dABCD* (for *Drosophila ABCD*), is 53% identical and 71% similar to human *ABCD1* (Fig. S1). Expression studies (Chintapalli et al., 2007) reveal moderate to high levels of *dABCD* in the adult head, consistent with a role for *dABCD* in the maintenance of CNS health in flies. As no genetically defined lesions for *dABCD* exist, we employed a short-hairpin microRNA from the VALIUM20 collection (*dsRNA-HMS02382*; confirmed bioinformatically to have no off-target effects) to target *dABCD* for studies of gene function. We found that *dABCD^tub>dsRNA^* transgenics survive to adulthood, but suffer from neurodegeneration. Specifically, *dABCD^tub>dsRNA^* flies exhibit a brain phenotype indistinguishable from that of animals homozygous for amorphic alleles of the *bgm-* and *dbb-*encoded *Drosophila* long/very-long-chain ACSs – that being an age-dependent retinal disorganization that is distinguished by retinal holes and pigment cell loss (Fig. 1A,B,F). Previous reports (Min and Benzer, 1999; Sivachenko et al., 2016) have validated both *bgm* and *dbb* as ALD-like models of neurodegeneration. However, the utility of these lines as disease models is now further bolstered by their shared loss-of-function phenotype with *dABCD*. Moreover, it is clear that *ABCD1* should be added to the growing list of human neurodegenerative-disease-related genes with functional homologs in *Drosophila* (Chien et al., 2002).

Having established here and elsewhere (Sivachenko et al., 2016) that both neurons and glia die in *Drosophila* ABCD and ACS models of neurodegeneration, we next sought to determine the cell-type-specific requirements for the *Drosophila*-encoded FA transporter and synthetases. To this end, we used *elav* (neuronal), *sim* (embryonic midline glial and adult neuronal) and *repo* (glial) drivers (FlyBase, 2003) to mediate cell-type-specific expression of dsRNA targeting *dABCD*. Neuronal disruption of *dABCD* in *dABCD^elav>dsRNA^* and *dABCD^sim>dsRNA^* transgenics recapitulates retinal defects seen in *dABCD^tub>dsRNA^* animals; in contrast, no defects result from glial-specific disruption in *dABCD^repo>dsRNA^* transgenics (Fig. 1C-E,K). Complementing this analysis is our study of the effects of cell-type-specific expression of a *Drosophila bgm^+^* transgene in a *bgm^1^* null mutant background. Both ubiquitous and neuronal *bgm^+^* expression rescue age-dependent neurodegeneration in *bgm^1^* mutants, although glial expression does not (Fig. 1F-J,L). Our observation of identical tissue-specific effects for *dsRNA-HMS02382*-mediated *dABCD* gene disruption and *bgm^+^* gene rescue is consistent with bioinformatic exclusion of off-target effects for *dsRNA-HMS02382*. Moreover, results from targeted disruption (*dABCD*) and rescue (*bgm*) studies show that the primary site of ABCD/ACS function in the *Drosophila* model of ALD-like neurodegeneration is the neuron and point to this cell type as the optimal target for prevention of neurodegeneration in ACS and ABCD fly models, and, by extension, for ALD therapy in humans.

### Gene-environment interactions modulate ALD penetrance and expressivity

**Fig. 2.**
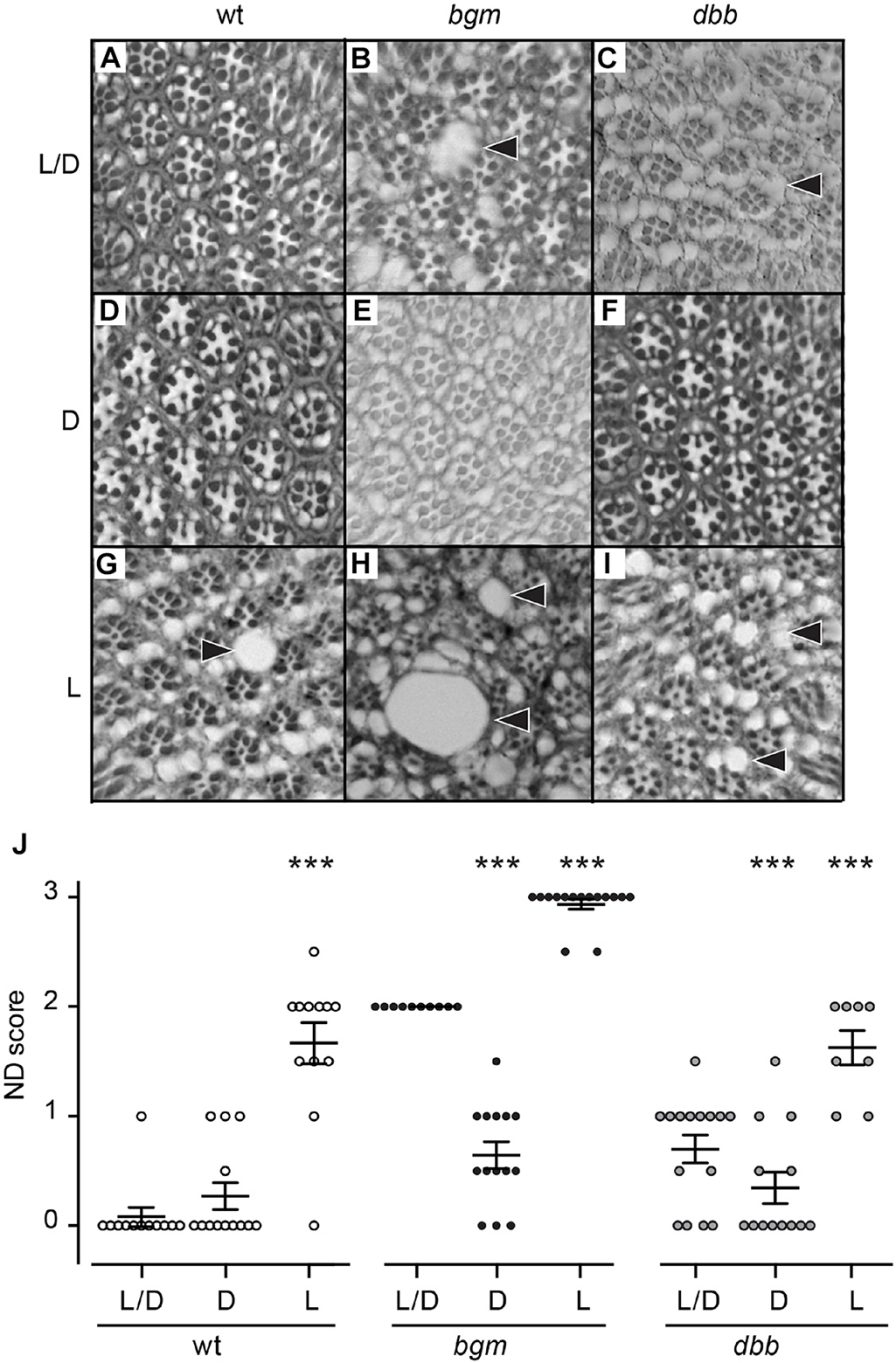
**Light-dark cycles modulate neurodegeneration in *bgm* and *dbb* single mutants.** (A-C) Cross-sections of retinas from wild-type (wt), *bgm* and *dbb* animals raised in standard 12-h light/dark cycles (L/D), with *bgm* and *dbb* mutants showing normal patterns of degeneration. (D-F) Constant dark (‘D’) alleviates degeneration, whereas (G-I) constant light (‘L’) exacerbates degeneration. (J) Blinded quantification of neurodegenerative (ND) phenotypes. Scores were compared using one-way ANOVA with Dunnett's. Data points represent biological replicates and are presented with means±s.e.m. ***P<0.001. Arrowheads point to areas of retinal degeneration.

As is true for ALD patients, neurodegeneration in *bgm*, *dbb* and *dABCD* flies is incompletely penetrant and variably expressed (Sivachenko et al., 2016; see also Fig. 1). The origin of this variability is so far unknown, although multiple reports have pointed to an environmental interaction (Weller et al., 1992; Raymond, et al., 2010; Sivachenko et al., 2016). Thus, to examine environmental contributions to phenotype, we examined responses of *bgm^1^* and *dbb^1^* amorphs to environmental stress in the form of light manipulation (Johnson et al., 2002). In contrast to animals raised in normal light/dark conditions (12 h light:12 h dark), animals raised in constant dark (24 h dark) exhibit significantly less neurodegeneration; in the case of *dbb^1^*, neurodegeneration appears to be blocked entirely (Fig. 2A-F,J). In complementary constant light conditions, we observed a significant exacerbation of neurodegeneration in all backgrounds, including the wild type (Fig. 2G-J). Finally, in defining constant-light-induced neurodegeneration in wild-type animals as baseline, we determined that enhanced neurodegeneration in *bgm^1^* and *dbb^1^* animals is synergistic (Fig. 2J). Thus, environmental stress in the form of light modifies neurodegenerative phenotypes in *bgm* and *dbb* models of neurodegeneration, demonstrating that gene-environment interactions can modulate penetrance and expressivity of neurodegenerative phenotypes.

### Product insufficiency is causative of ALD

**Fig. 3.**
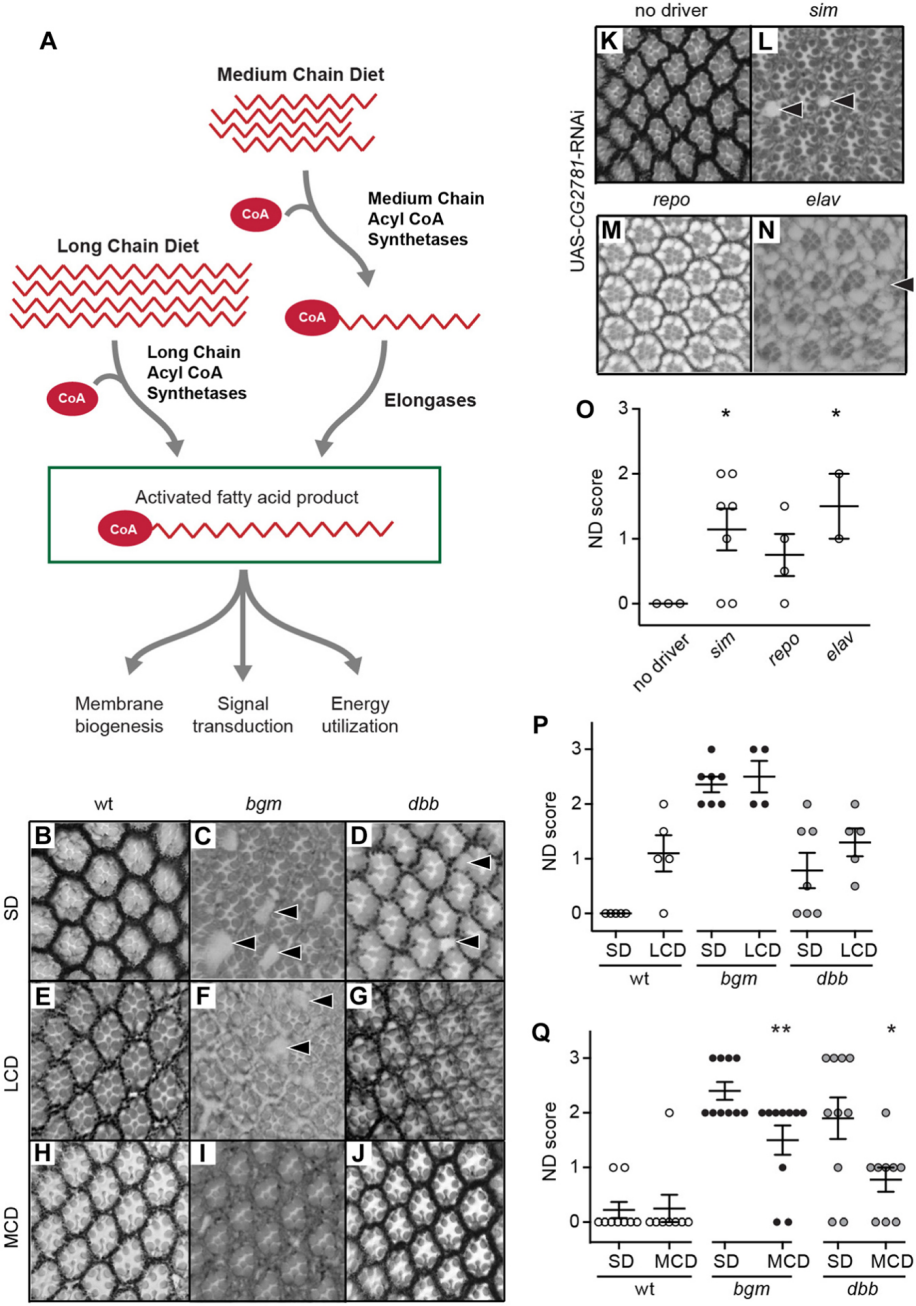
**Neurodegeneration in *bgm* and *dbb* animals is caused by a lack of activated fatty acid (FA) product(s).** (A) Two pathways, one depending on long- and the other on medium-chain FAs, converge to produce activated long- and very-long-chain fatty acid products. (B-D) Cross-sections of retinas from wild-type (wt), *bgm* and *dbb* animals fed a standard diet (SD). (E-G) Retinas from wt, *bgm* and *dbb* animals fed a diet enriched in long-chain FAs (LCD) are indistinguishable from genotypically matched animals fed SD. (H-J) Diets rich in medium-chain fatty acids (MCD) suppress neurodegeneration in *bgm* and *dbb* mutants. (P,Q) Blinded scoring of neurodegeneration in animals fed SD compared with those fed with LCD or MCD. (K-N) Cross-sections of retinas from animals with and without tissue-specific expression of an RNAi targeting the *Drosophila* elongase *CG2781*. (O) Blinded scoring of neurodegenerative (ND) phenotypes in the RNAi experiment. All scores were compared by one-way ANOVA with Dunnett's test. Data points represent biological replicates and are presented with means±s.e.m. **P<0.01, *P<0.05. Arrowheads point to areas of retinal degeneration.

Despite accumulation of VLCFAs (ACS and ABCD1 substrates) in ALD patients, it is still debated whether this marker of disease is also causative of disease, and whether accumulating VLCFAs should serve as a therapeutic target (Raymond et al., 1999). Here, we consider the alternative possibility – that it is the absence of activated VLCFAs and/or their metabolic products that is causative of disease. As a first step toward disease therapeutics and in our first direct test of the lack of product hypothesis for ALD, we fed *bgm^1^* and *dbb^1^* animals a diet high in medium-chain fatty acids [7% coconut oil (Birse et al., 2010)] from day 0 (d0) to day 20 (d20) post-eclosion, anticipating that bypass of the genetic block to activating long-chain FAs (LCFAs)/VLCFAs in *bgm* and *dbb* mutants via the elongase pathway might suppress neurodegeneration (Fig. 3A). Indeed, supplementation of *bgm^1^* and *dbb^1^* mutant diets with medium-chain FAs significantly reduces retinal defects observed at d20 post-eclosion in both *bgm^1^* and *dbb^1^* animals (Fig. 3B-D,H-J,Q). Second, in anticipation that increasing LCFAs/VLCFAs will enhance neurodegeneration if accumulating precursors are toxic (see Fig. 3A), we examined neurodegeneration in *bgm^1^* and *dbb^1^* animals fed a diet high in LCFAs (described in Carvalho et al., 2012). We found no changes in neurodegeneration in animals fed LCFA-enriched diets (Fig. 3E-G,P). That mutant neurodegenerative phenotypes were rescued by medium-chain-FA dietary supplementation and not exacerbated by LCFA dietary supplementation points to product loss as being causative of neurodegenerative disease.

### A parallel route to VLCFA production (the elongase pathway) is required for CNS health and maintenance

Given the deficiencies of Lorenzo's oil in the treatment of ALD patients (Moser, 1999; van Geel et al., 1999; Zinkham et al., 1993) and as an extension of our medium-chain dietary treatment results in *bgm* and *dbb* flies, we next tested whether disruption of the FA elongase pathway that uses activated medium-chain FAs to produce activated LCFAs is associated with neurodegeneration (see Fig. 3A). Using BLASTp, we identified four genes encoding *Drosophila* elongases [there are seven in humans (Jump, 2009)], but only one, *CG2781* (44-56% identical to *ELOVL1*, *7* and *4* and henceforth identified as *dELOVL* for *Drosophila ELOVL*; Fig. S2), is expressed in a spatial and temporal manner analogous to *dABCD* and predicative of a role in neuronal health and maintenance (Chintapalli et al., 2007). There are no genetically defined *dELOVL* mutants; thus, as we did previously for *dABCD1*, we used the binary UAS-GAL4 system in combination with RNAi-mediated gene disruption methods. We used two reagents to disrupt *ELOVL* gene function and thereby assess its function in CNS health and maintenance*.* The first, *dsRNA-HMC03112*, is from the VALIUM20 TRiP collection and has been confirmed bioinformatically to have no off-target effects. The second, *dsRNA-GD16713*, is from the Vienna *Drosophila* Resource Center. While ubiquitous *dELOVL* disruption (in *dELOVL^tub>dsRNA-HMC03112^* transgenics) leads to lethality before eclosion (data not shown) and points to an early essential role for *dELOVL*, specific neuronal knockdown of *dELOVL* (in *dELOVL^sim>dsRNA-HMC03112^* and *dELOVL^elav>dsRNA-HMC03112^*) leads to neurodegeneration. Animals exhibit retinal defects, including holes and lost pigment cells, phenotypes replicating those that we have observed in *bgm-*, *dbb-* and *dABCD1*-deficient animals. In an extension of the analysis, targeted disruption of *dELOVL* in glia (in *dELOVL^repo>dsRNA-HMC03112^* transgenics) does not produce degenerative phenotypes (Fig. 3K-O). Thus, cell-type specificity for all pathway components in VLCFA metabolism (ACSs, elongases and transporters) is neuronal. Despite a high background for leaky *dsRNA-GD16713* expression that causes death in the absence of a GAL4 driver, we reproduced all *dsRNA-HMC03112* qualitative results using *dsRNA-GD16713* (data not shown). Together, these data show that product loss is causative of disease and that mutations in genes encoding elongases and medium-chain FA acyl-CoA synthetases should be considered candidate ALD-causing disease genes, as well as targets for therapeutic options.

## DISCUSSION

ALD is a progressive neurodegenerative disease, with the most severe form claiming the lives of school-age boys. Although *ABCD1*, the gene responsible for the most common form of ALD (X-ALD), has been cloned, the etiology of the disease has remained elusive and there are still no satisfactory treatments or cures (for review see Gordon et al., 2014). Using *in vivo* models of neurometabolic disease, we here provide new insights into human ALD. Our demonstration that mutations in long- and medium-chain FA metabolic pathways in *Drosophila* yield shared loss-of-function neurodegenerative phenotypes extends a single-gene association (*ABCD1*) for ALD to a pathway association (lipid metabolism). Importantly, this more expansive view of ALD offers a possibility of diagnosis to some of the 50% of leukodystrophy patients with undiagnosed conditions (Gordon et al., 2014). In addition, our demonstration that neurodegeneration in fly models of ALD does not result from a buildup of FA precursors, but instead is caused by a lack of activated FA product, shifts our understanding of ALD etiology and is expected to have profound effects on the design of effective therapeutics. Indeed, our data indicate that a diet high in medium-chain FAs provides a potential therapeutic approach for leukodystrophy patients with ACS mutations (Sivachenko et al., 2016). At the very least, our study validates the continued search for remedies other than Lorenzo's oil for the treatment of X-ALD (Eichler et al., 2017; Kolata, 2017).

Although hundreds of *ABCD1* alleles are associated with ALD, no genotype-phenotype correlations have emerged. Likewise, inheritance of the same allele within kindreds (or even an allele common to monozygotic twins) can lead to different disease phenotypes (Berger et al., 1994; Korenke et al., 1996). Thus, it seems clear that gene-environment interactions modulate ALD penetrance and expressivity. Gene-environment interactions extend to other players in the lipid metabolic pathway associated with ALD as well. In this regard, each of the young brothers in a recently described Utah leukodystrophy family harbors an allele pair associated with two incompletely penetrant conditions (*PRRT2*/childhood epilepsy and *Slc27a6*/leukodystrophy). Only the younger brother, however, exhibits both seizure and leukodystrophy phenotypes (Sivachenko et al., 2016).

Our finding that environmental stress in the form of light modifies neurodegenerative phenotypes in *bgm* and *dbb* models of neurodegeneration provides direct evidence for a gene-environment interaction that modulates penetrance and expressivity of neurodegenerative phenotypes (see Fig. 2). These data bolster our view that: (1) environmental stress in the form of seizure triggered neurodegeneration in a leukodystrophy patient with a predisposing ACS mutation (Sivachenko et al., 2016), and (2) traumatic brain injury triggered neurodegeneration in patients with catastrophic presentations of ALD (Raymond et al., 2010; Vawter-Lee et al., 2015; Weller et al., 1992). Moreover, as stress might precipitate a degenerative cellular phenotype when product generation is required to repair damaged or depleted cellular components, our *bgm/dbb* stress studies suggest that esterified LCFA and VLCFA products of Bgm and Dbb activity are required to prevent neurodegeneration, a hypothesis that contradicts the long-held view that precursor accumulation is causative of neurodegeneration in ALD patients.

In the *Drosophila bgm/dbb* model of neurodegeneration, inclusions in brain tissue and elevated levels of VLCFAs provide clear diagnostic markers of disease, and point to fly mutants as powerful *in vivo* models for testing the effects of FA dysregulation on neurodegeneration (Sivachenko et al., 2016). In humans, evidence for accumulating FAs as being causative of disease comes primarily from tissue culture studies, where accumulating FAs can lead to cell death (Hein et al., 2008; Reiser et al., 2006; Ulloth et al., 2003). Contradicting this view, however, are data from *ABCD1* hemizygotes showing that, although all individuals harboring mutant alleles of *ABCD1* exhibit similar significant increases in their circulating VLCFA levels, these correlate with neither the development of disease nor the timing of disease onset (Dubey et al., 2005; Raymond et al., 1999). Moreover, some recipients of hematopoietic stem cell gene therapy show improvement in neurological symptoms despite plasma VLCFA concentrations remaining significantly high (Cartier et al., 2009). Understanding disease etiology represents an essential first step in providing appropriate therapies. The current therapeutic option for the most severe ALD cases is bone marrow transplant; however, the procedure itself carries substantial risk and is not always successful (Berger et al., 2010; Gordon et al., 2014). Another touted therapy called Lorenzo's oil is thought to target accumulated VLCFAs but remains a controversial option, with most agreeing that it is ineffective (Aubourg et al., 1993; Berger et al., 2010; Gordon et al., 2014).

Here, we demonstrate that *bgm* and *dbb* neurodegenerative phenotypes are rescued by medium-chain FA dietary supplementation, while being unaffected by LCFA (see Fig. 3). Together, data from these complementary studies identify product loss as causative of neurodegenerative disease. Indeed, end products of peroxisomal metabolism (including both glycerolipids and plasmalogens) constitute more than 80% of the phospholipid content of brain white matter (Schrader and Fahimi, 2008). Additionally, our results are the first to highlight potential for the activated medium-chain elongation pathway as an alternate route to production of missing VLCFA product(s) in ACS mutants. Of course, dietary supplementation is not necessarily expected to rescue *ABCD* *Drosophila* mutants (or to provide therapeutic value to X-ALD patients) because *dABCD/ABCD* functions at the intersection of the long/very-long- and medium-chain lipid metabolic pathways (see Fig. 3A). This said, our prior association of the *SLC27a6*-encoded ACS with ALD (Sivachenko et al., 2016) suggests that there are forms of the disease that are likely to be alleviated by dietary supplementation with medium-chain FAs.

Finally, over half of leukodystrophies remain undiagnosed. Our identification of the elongase pathway identifies new candidates for disease genes. At the biochemical level, fatty acyl-CoA chain elongation involves the addition of two carbon units to an existing fatty acyl-CoA, thereby bypassing a requirement for VLCFA activation by long- and very-long-chain ACSs (Beaudoin et al., 2002). Elongases have been implicated in ALD etiology, as enzyme levels are increased in induced pluripotent stem cell (IPSC)-derived brain cells from ALD patients (Baarine et al., 2015). Although increased elongase levels have been interpreted to mean that elongase function might exacerbate disease in patients, functional tests have yet to be undertaken and another interpretation of the data is that elongase levels are upregulated in *ABCD1* mutants as an alternate route to the production of essential activated VLCFAs. Interestingly, in humans, the longest VLCFA species are found only in tissues expressing elongases, namely the retina, brain and testis, all three of which are key tissues affected in ALD (Agbaga et al., 2010; Ahmad et al., 2009).

In summary, ALD in its most severe form results in acute and rapidly progressing degeneration of brain white matter and leads to death within a few years of diagnosis. The etiology of ALD has remained poorly understood, and there is still no treatment for this condition. ALD has long been thought to result from a buildup of VLCFAs in the brains of affected individuals. We used the *Drosophila* model of neurodegeneration to show that this long-held view is incorrect. While accumulating VLCFAs are indeed a marker of neurodegenerative disease in both flies and humans, we show that it is the absence of activated VLCFAs and or/their metabolic products that is causative of disease in the fly model. Our studies contribute to the fields of ALD and neurodegenerative disease in three major ways: (1) we show that activated VLCFAs and/or their products are necessary for neuronal health and maintenance; (2) we identify new candidate ALD-disease-causing genes, and (3) we show that a diet high in medium-chain FAs shows promise as a potential therapeutic approach for patients with neurometabolic degenerative disease.

## MATERIALS AND METHODS

### *Drosophila* stocks

*Gal4* lines used to drive targeted transgene expression include *repo-Gal4* (BL#7415), *elav-Gal4* (BL#8760), *sim-Gal4* (BL#9150) and *DJ667-Gal4* (BL#8171); *tubulin*-*Gal4* (*w^1118^; P{tubP-gal4}LL7/TM3, P{Dfd-GMR-nvYFP}3 Sb1*) was the gift of Mark Metzstein (University of Utah, Salt Lake City, UT). The *bgm^1^* and *dbb^1^* mutants have been described (Min and Benzer, 1999; Sivachenko et al., 2016). dsRNA lines targeting *dABCD1* and *dELOVL* were obtained from the Transgenic RNAi Project at Harvard Medical School (#41984 and #50710, respectively); an additional dsRNA line targeting *dELOVL* was obtained from the Vienna *Drosophila* Resource Center (v102543). Unless otherwise noted, all flies were raised on a standard cornmeal diet at 25°C with 12-h light:dark cycles.

For tissue-specific expression of *bgm*, its coding sequence was inserted into pFLAG-CMV-5a (Sigma #E7523) using primers 5′-ATAAAGCTTATGTCCACGATAGACGCGCTC-3′ and 5′-GCGGTACCGGCATATAGTTTCTCGATCTC-3′. The tagged version of the gene was subsequently inserted into the *Drosophila* expression vector pUAST using primers 5′-AATGGGCGGTAGGCGTGTACG-3′ and 5′-AATCTAGACTCGAGATTAGGACAAGGCTGGTGGGC-3′. pUAS-bgm-FLAG was sequence verified and co-injected with transposase Δ2-3 into dechorionated *nosΦC31; +; vk27* embryos 1-2 h after egg lay. G0 injected animals were mated to *w^1118^*, and progeny were screened for transformed germlines based on eye color. The *UAS-bgm-FLAG* line used for our studies is homozygous viable, with the transgene insertion on the second chromosome.

### Diet and light manipulations

Medium- and long-chain diets were prepared as previously described (Birse et al., 2010; Carvalho et al., 2012). Adult males were collected within 24 h of eclosion and maintained on prescribed diets until sacrifice 20-22 days post-eclosion. For light manipulations, males were isolated within 48 h of eclosion and habituated in 24 h light, 12 h light/dark, or 24 h dark cycles in a temperature- and humidity-controlled room until sacrifice at 20-22 days post-eclosion.

### Aging and histology

Heads from adult *Drosophila* males were prepared, sectioned and imaged as previously described (Sivachenko et al., 2016). Samples were scored blindly in three to five serial sections for each animal. The degree of retinal degeneration was scored qualitatively as 0 for normal appearance, 1 for mild tissue loss, 2 for moderate degeneration and 3 for severe degeneration (Cao et al., 2013). Data were analyzed by ANOVA and Welch two-sample *t*-tests. Data were analyzed using GraphPad Prism software (GraphPad Software).

## Supplementary Material

Supplementary information

First Person interview
