# Supplementary material for: Etiology and treatment of adrenoleukodystrophy: new insights from Drosophila
Source: Dis Model Mech. 2018 Jun 15;11(6):dmm031286. doi: 10.1242/dmm.031286 (PMC6031365; doi:10.1242/dmm.031286)
Supplement: Supplementary information [file dmm-11-031286-s1.pdf]

```
Hsap ABCD1 aligned with Dmel cg2316:

Dmel cg2316    1    MS-VLSKYVDRIAEKCEHNGFTKHAFSYALVTSAILALTIKVTIPYVKNVNTTSSVRTQKGKTNGQLSPSTRDSSEEDFK   79
Hsap ABCD1     1    MTHMLNAAADRV--KWTRSSAAKRA---ACLVAAAYAL--KTLYPIIGKRLKQSG----HGKKKAAAYPAAENT------   63

Dmel cg2316    80   LAEAEKLLVAQQLKKKATNNLVEPGLNKEFLKHLQMLAKIMIPQAFCYETGLLSVHTFCLISRTFLSIYVAALEGALVKF   159
Hsap ABCD1     64   ----EILHCTETICEKPS-----PGVNADFFKQLLELRKILFPKLVTTETGWLCLHSVALISRTFLSIYVAGLDGKIVKS   134

Dmel cg2316    160  IVRKDIKQFALVLLKWFGIAIPATFVNSMIRFLESKLSLAFRTRLVRHSYRLYFKNQNYYRVSNLDGRIENADHRLTEDI   239
Hsap ABCD1     135  IVEKKPRTFIIKLIKWLMIAIPATFVNSAIRYLECKLALAFRTRLVDHAYETYFTNQTYYKVINMDGRLANPDQSLTEDI   214

Dmel cg2316    240  SVFANSVAHLYSSLTKPCFDLMLIGLALMRSSKKMKANIITGPALSIGVIALTAHILRIVSPKFGQLVSEEANRYGYLRH   319
Hsap ABCD1     215  MMFSQSVAHLYSNLTKPILDVMLTSYTLIQTATSRGASPIGPTLLAGLVVYATAKVLKACSPKFGKLVAEEAHRKGYLRY   294

Dmel cg2316    320  IHSRIITNAEEIAFYGGHKVEMQQLRQAYNRLVNQMTTIFNQKLWFIMLEQFFMKYVWSGTGMIMVSLPILTGSDVGLGT   399
Hsap ABCD1     295  VHSRIIANVEEIAFYRGHKVEMKQLQKSYKALADQMNLILSKRLWYIMIEQFLMKYVWSSSGLIMVAIPIITATGFADG-   373

Dmel cg2316    400  VPNTAISESRVSERTQYLTTARNLLISAADAIERLMSSYKEIVSLAGYTFRVAGMMDVFEETALGVYCKTSVM---ESNQ   476
Hsap ABCD1     374  --EDGQKQVMVSERTEAFTTARNLLASGADAIERIMSSYKEVTELAGYTARVYNMFWVFDEVKRGIYKRTAVIQESESHS   451

Dmel cg2316    477  SNGI-IEFRNGKPIA-KGRIIYSDDPKNMSISLRAVPVVTPNCDIVVPKLTLCIEPGVHLLITGPNGCGKSSLFRILSGL   554
Hsap ABCD1     452  KNGAKVELPLSDTLAIKGKVIDVDH----GIICENVPIITPAGEVVASRLNFKVEEGMHLLITGPNGCGKSSLFRILSGL   527

Dmel cg2316    555  WPIYAGELHIPRPVKDVPCMFYIPQRPYMSIGSLCDQIIYPDTREDMKRKHITENELRSILKMVSLEHIAQRDS-FDVVR   633
Hsap ABCD1     528  WPVYEGVLYKPPPQH----MFYIPQRPYMSLGSLRDQVIYPDSVDDMHDKGYTDQDLERILHNVHLYHIVQREGGWDAVM   603

Dmel cg2316    634  DWKDILSGGEKQRMAIARLFYHRPRYALLDECTSAVSIDVESSIYEIAKGMGITLLTITHRPTLWKYHTHILEFDGLGNW   713
Hsap ABCD1     604  DWKDVLSGGEKQRMGMARMFYHKPKYALLDECTSAVSIDVEGKIFQAAKGAGISLLSITHRPSLWKYHTHLLQFDGEGGW   683

Dmel cg2316    714  QFRKMN-------SDEEQK-----------GQFLS----------------------   730
Hsap ABCD1     684  RFEQLDTAIRLTLSEEKQKLESQLAGIPKMQQRLNELCKILGEDSVLKTIKNEDETS   740

RED = identical residues
BLUE = conserved residues
```

Figure S1. Amino acid alignment of human ABCD1 and Drosophila CG2316 shows high levels of conservation. Sequences were aligned using COBALT software through NCBI.

Disease Models & Mechanisms • Supplementary information

```
Dmel cg2781 aligned with Hsap ELOVL7, ELOVL1, and ELOVL4:

Dmel cg27811     MDYLTM------------------FYDGWRDLMDnkSDPRTRDYPLMSSPF--PTIAISLTYaYIVKVLGPKLMENRKPF  60
Hsap ELOVL71     MAFSD------------LTSRTVHLYDNWI--KD--ADPRVEDWLMSSPL-PQTILLGFYV-YFVTSLGPKLMENRKPF  62
Hsap ELOVL11     M------------------EAVVNLYQEVM--KH--ADPRIQGYPLMGSPLlMTSILLT-YV-YFVLSLGPRIMANRKPF  56
Hsap ELOVL41     MGLLDSepgsvlnvvstALNDTVEFYRWTWSI----ADKRVENWPLMQSPW--PTLSISTLY-LLFVWLGPKWMKDREPF  73

Dmel cg278161    ELRKVLIVYNAAQVIFSAWLFYESCIGGWLNGYNLRCEPVNYSYSPKAIRTAEGCWWYYFSKFTEFFDTFFFVMRKRYDQ  140
Hsap ELOVL763    ELKKAMITYNFFIVLFSVYMCYEFVMSGWGIGYSFRCDIVDYSRSPTALRMARTCWLYYFSKFIELLDTIFFVLRKKNSQ  142
Hsap ELOVL157    QLRGFMIVYNFSLVALSLHIVYEFLMSGWLSTYTWRCDPVDYSNSPEALRMVRVAWLFLFSKFIELMDTVIFILRKKDGQ  136
Hsap ELOVL474    QMRLVLIIYNFGMVLLNLFIFRELFMGSYNAGYSYICQSVDYSNNVHEVRIAAALWWYFVSKGVEYLDTVFFILRKKNNQ  153

Dmel cg2781141   VSTLHVIHHGIMPVSVWWGVKFTPGGHSTFFGFLNTFVHIFMYAYYMLAAMGPKVQKYLWWKKYLTVMQMIQFVLVMVHS  220
Hsap ELOVL7143   VTFLHVFHHTIMPWTWWFGVKFAAGGLGTFHALLNTAVHVVMYSYYGLSALGPAYQKYLWWKKYLTSLQLVQFVIVAIHI  222
Hsap ELOVL1137   VTFLHVFHHSVLPWSWWWGVKIAPGGMGSFHAMINSSVHVIMYLYYGLSAFGPVAQPYLWWKKHMTAIQLIQFVLVSLHI  216
Hsap ELOVL4154   VSFLHVYHHCTMFTLWWIGIKWVAGGQAFFGAQLNSFIHVIMYSYYGLTAFGPWIQKYLWWKRYLTMLQLIQFHVTIGHT  233

Dmel cg2781221   FQLFFKNDCNYPI-GFAYFIGAHAVMFYFLFSNFYKRAYV--KRDGKDKASVKANGhANGHVKALKDGDVA---PTSNGQ  294
Hsap ELOVL7223   SQFFFMEDCKYQFPVFACIIMSYSFMFPLLFLHFWYRAYT--KGQRLPKT-VKN-----GTCKNKDN-------------  281
Hsap ELOVL1217   SQYYFMSSCNYQYPVIIHLIWMYGTIFFMLFSNFWYHSYT--KGKRLPRA-LQQNG-APGIAKVKAN-------------  279
Hsap ELOVL4234   ALSLY-TDCPFPK-WMHWALIAYAISFIFLFLNFYIRTYKepKKPKAGKTAMNGIS-ANGVSKSEKQLMIEngkKQKNGK  310

Dmel cg2781295   ANGFhntfskfttdmcnpalnsstrqrvlvnagnk  329
Hsap ELOVL7      -----------------------------------
Hsap ELOVL1      -----------------------------------
Hsap ELOVL4311   AKGD-------------------------------  314

RED = identical residues
BLUE = conserved residues
```

Figure S2. Amino acid alignments of *Drosophila* elongase CG2781 and three human elongases (ELOVL1,4,and 7). Sequences were aligned using COBALT software through NCBI.

Disease Models & Mechanisms • Supplementary information
