# Supplementary material for: Etiology and treatment of adrenoleukodystrophy: new insights from Drosophila
Source: Dis Model Mech. 2018 Jun 15;11(6):dmm031286. doi: 10.1242/dmm.031286 (PMC6031365; doi:10.1242/dmm.031286)
Supplement: First Person interview [file dmm-11-031286-s2.pdf]

FIRST PERSON

First person – Hannah Gordon

First Person is a series of interviews with the first authors of a selection of papers published in Disease Models & Mechanisms, helping early-career researchers promote themselves alongside their papers. Hannah Gordon is first author on 'Etiology and treatment of adrenoleukodystrophy: new insights from *Drosophila*', published in DMM. Hannah conducted the research in this article while a PhD student in the lab of Anthea Letsou at the University of Utah, Salt Lake City, USA, but is now a postdoc in Kristen Kwan's lab at the same institute, investigating signaling pathways and morphogenesis in eye development.

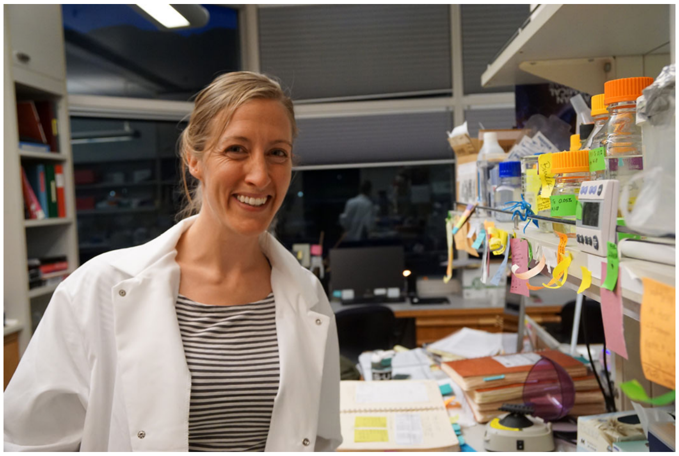

Hannah Gordon

How would you explain the main findings of your paper to non-scientific family and friends?

This paper describes how disruption of a specific kind of fat metabolism results in neurodegeneration. Disruption of this metabolic pathway causes a human disease called adrenoleukodystrophy (ALD), which itself is fairly rare. However, the disease is unique in that it informs us on how the nervous system relies on certain fats to be healthy and maintain proper function, which can also apply to humans without the disease. In this paper, we used a fly model we previously developed (Sivachenko et al., 2016) to answer outstanding questions in the ALD field, namely, why does a block in this specific kind of fat metabolism make neurons die? For me, this was the most important and most interesting question we could use our fruit fly model to address. In humans and flies (and most living cells), fatty acids (a single unit of fat) need to be 'activated' by an enzyme inside of a cell before those fats can be modified by other enzymes and used for purposes such as building blocks for membranes or as fuel for energy. Only a few enzymes perform this activation function and in humans and flies with ALD, the function of one of these enzymes is disrupted. With this enzyme activity reduced (or completely off), two things happen in the cell: 1) an accumulation of fatty acid precursor and 2) a lack of activated fatty acid product. We wanted to know which of these two consequences of enzyme disruption resulted in neurodegeneration. We tested this by supplying mutant flies with fatty acids from which they could produce a missing product, and this alleviated their neurodegeneration. We also found that if we blocked a different pathway that produces the same activated fatty acid product, in an otherwise healthy fly we could cause neurodegeneration. In addition to suggesting a much-needed potential therapy for ALD patients, this paper also identifies additional genes that might be causative of other undiagnosed leukodystrophies and sheds light on the inner workings of the nervous system and its dependence on (or sensitivity to) specific kinds of fats for overall health.

"Validating our fly model of ALD in our previous DMM paper provided a platform to take this model one step further"

Hannah Gordon's contact details: Department of Human Genetics, University of Utah, Salt Lake City, UT 84112, USA.

E-mail: hannah.gordon@utah.edu

What are the potential implications of these results for your field of research?

Before this paper, the main hypothesis in the field was that when you block this step in fat metabolism, a build-up of fat precursor occurred and that this build-up was toxic to the cells, resulting in neuron death. Multiple observations over decades, however, suggested this wasn't the whole story, but there was no *in vivo* system available that allowed an uncoupling of the build-up of precursor from the lack of activated fatty acid product. Validating our fly model of ALD in our previous DMM paper provided a platform to take this model one step further and with various methods, uncouple the fatty acid precursor and product so they could be tested independently. I think gaining an understanding of how specific kinds of fats are used by the nervous system is fascinating on its own, but pinning down the cause of death for neurons is crucial when we think about therapeutic approaches. For example, if the build-up of precursor is toxic, our therapeutic approach would be to find ways to prevent or remove that build-up (which has been attempted in the past and failed). However, if the lack of activated product is causing neuron death, our therapeutic approach would be quite the opposite, where we try to find ways to supply the cells with the fatty acids they need. I think this fundamental shift in therapeutic approach is the biggest impact of our results.

What are the main advantages and drawbacks of the model system you have used as it relates to the disease you are investigating?

Having a fruit fly model of this disease is really a scientist's dream come true. Flies have a nervous system that is much simpler than our nervous system but if you zoom in to the cellular level, a fly neuron functions almost indistinguishably from that of a human neuron. Zoom out, and that fly neuron resides in a much simpler organism that is easier to study than humans. Flies also offer so many genetic tools to test complex ideas about how the nervous system functions (and more of these tools are being generated every month). Probably the biggest drawback is that it's sometimes hard to convince people

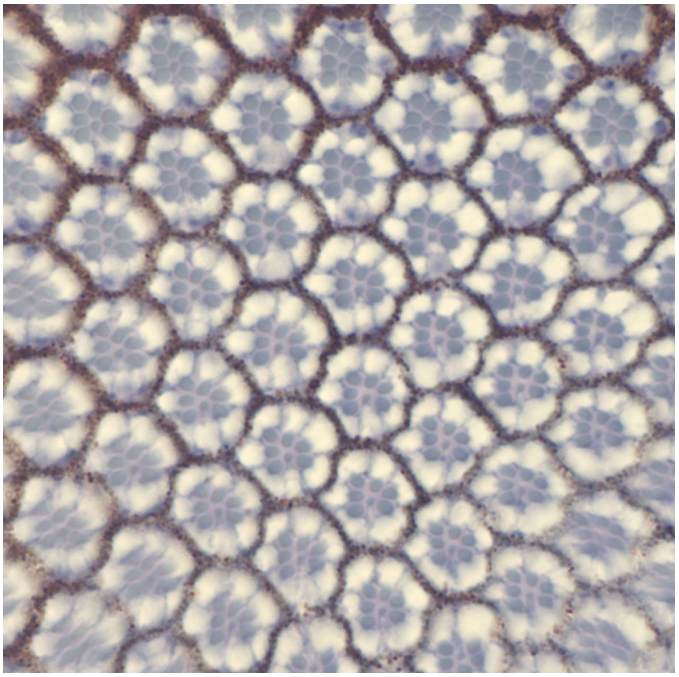

Cross section of a fruit fly eye stained with Toluidine Blue shows the tight organization of photoreceptors in the nervous system (blue). Disorganization of these cells is a sensitive way to test for neurodegeneration.

of the strengths of this model organism (and other model organisms, for that matter) and thus it can be difficult to obtain funding for such productive research, despite these model organisms proving their value many, many times over.

"Probably the biggest drawback is that it's sometimes hard to convince people of the strengths of this model organism (and other model organisms, for that matter)"

What has surprised you the most while conducting your research?

The rescue with medium-chain fatty acids really surprised me! This idea that neurodegeneration could also be caused by a lack of fatty acid product really stemmed from looking at multiple lines of evidence that, despite a lot of work from very dedicated people, we didn't have the whole story right. It was a fundamentally simple hypothesis, so we thought that if we could do it right, why not test it?

Describe what you think is the most significant challenge impacting your research at this time and how will this be addressed over the next 10 years?

One main conclusion of our paper is that a particular kind of fatty acid might be necessary for maintaining a healthy nervous system. If we think of fatty acids as links on a chain, they can be separated into classes based on chain length and the ones we think might be required for a healthy nervous system are the longest of these chains. That kind of fatty acid is fairly rare in the body and is most abundant in the testes, brain and retina. Because of their rarity, many studies that look at fatty acids gloss over this class, so we know less about what they are doing in the cell than the other classes of fatty acids. Biochemical techniques are changing, however, and many more researchers are using lipidomics to study all sorts of qualities of fatty acids. Ultimately, I'd love to know what that very-long-chain fatty acid is being used for in the cell and to answer this I'd love to see the development of more tools to study very-long-chain fatty acid biology.

What changes do you think could improve the professional lives of early-career scientists?

I would love to see the standard implementation of amenities like high-quality health insurance and a retirement plan at all universities, which might be necessary to retain the most talented postdoctoral scientists. Being a postdoc has traditionally been thought of as a transition period, which may be why those amenities were less emphasized as part of the postdoc position. However, competition for limited faculty positions is growing, requiring more time as a postdoc to meet the conditions for these positions. From what I've observed, one major determining factor for whether talented individuals stay in academia is whether they can afford to spend years working well below their post-degree earning potential, which can be heavily influenced by socioeconomic background. On top of this is that an individual's 20s and 30s (the age range of most early career scientists) represent a critical earning period in their lives. I was introduced to academia as a place for anyone seeking the pure endeavors of knowledge, leading to a growing community of diverse and passionate individuals of all ethnicities. I'd love to see academia challenge itself to continue broadening this acceptance through support of different socioeconomic backgrounds at the postdoctoral level. I think the positive effects of these efforts could be socially far-reaching in 1) making science more accessible for more people and 2) casting aside the all-too-common association of science as an endeavor only of the elite.

What's next for you?

Since finishing my PhD I have moved to a completely new organism and field and I am so far enjoying all of the new adventures my research has brought me. I am working hard to develop zebrafish as a model organism for a new field of research. With its ever-growing genetic toolkit and optical transparency, I'm really excited about the contributions this tiny vertebrate can add to understanding fundamental biology as well as human health and disease.

References

Gordon, H. B., Valdez, L. and Letsou, A. (2018). Etiology and treatment of adrenoleukodystrophy: new insights from *Drosophila*. *Dis. Model. Mech.* **11**: doi:10.1242/dmm.031286,

Sivachenko, A., Gordon, H. B., Kimball, S. S., Gavin, E. J., Bonkowsky, J. L. and Letsou, A. (2016). Neurodegeneration in a *Drosophila* model of adrenoleukodystrophy: the roles of the Bubblegum and Double bubble acyl-CoA synthetases. *Dis. Model. Mech.* **9**: 377-387, doi: 10.1242/dmm.022244.
